# Greater efficacy of atorvastatin versus a non-statin lipid-lowering agent against renal injury: potential role as a histone deacetylase inhibitor

**DOI:** 10.1038/srep38034

**Published:** 2016-11-30

**Authors:** Ravi Shankar Singh, Dharmendra Kumar Chaudhary, Aradhana Mohan, Praveen Kumar, Chandra Prakash Chaturvedi, Carolyn M. Ecelbarger, Madan M. Godbole, Swasti Tiwari

**Affiliations:** 1 Department of Molecular Medicine & Biotechnology, Sanjay Gandhi Post Graduate Institute of Medical Sciences, Lucknow, India; 2 Department of Hematology, Sanjay Gandhi Post Graduate Institute of Medical Sciences, Lucknow, India; 3 Department of Medicine, Georgetown University, Washington DC, USA

## Abstract

Statins, 3-hydroxy-3-methyl-glutaryl-coenzyme A reductase inhibitors have been shown to improve diabetic nephropathy. However, whether they provide protection via Histone deacetylases (HDAC) inhibition is not clear. We conducted a comparative evaluation of Atorvastatin (AT) versus the non-statin cholesterol-lowering drug, Ezetimibe (EZT) on severity of diabetic nephropathy. Streptozotocin-treated male Wistar rats were fed a cholesterol-supplemented diet and gavaged daily with vehicle, AT or EZT. Control rats received normal diet and gavaged vehicle (n = 8–9/group). Diabetes increased blood glucose, urine albumin-to-creatinine ratio (ACR), kidney pathology and HDAC activity, and reduced renal E-cadherin levels. Both AT and EZT reduced circulating cholesterol, attenuated renal pathology, and did not lower blood glucose. However, AT was significantly more effective than EZT at reducing kidney pathology and HDAC activity. Chromatin immunoprecipitation revealed a significantly higher association of acetylated H3 and H4 with the E-cadherin promoter in kidneys from AT-, relative to EZT- or vehicle-treated rats. Moreover, we demonstrated a direct effect of AT, but not EZT, on HDAC-inhibition and, H3 and H4- acetylation in primary glomerular mesangial cells. Overall, both AT and EZT attenuated diabetic nephropathy; however, AT exhibited greater efficacy despite a similar reduction in circulating cholesterol. HDAC-inhibition may underlie greater efficacy of statins in attenuating kidney injury.

Diabetic Nephropathy (DN) has become a serious public health concern globally leading to end-stage renal failure in up to 30% of individuals suffering from diabetes. DN is characterized by progressive accumulation and deposition of extracellular matrix components, such as collagens and fibronectin, in the glomerular mesangium and tubulointerstitium. This leads to mesangial expansion. The later stages of interstitial expansion and glomerulosclerosis^1^ affect glomeruli with an increase in glomerular filtration rate, microalbuminuria, glomerular hypertrophy, and thickening of the glomerular basement membrane.

Dyslipidemia, often present in diabetes mellitus, has been suggested to play a pathogenic role in the progression of kidney disease in these patients^2^. Furthermore, reno-protective effects of statins have also been observed in large-scale human clinical trials, like WOSCOPS, CTT, DALI, CARDS and TNT^3^,^4^,^5^,^6^,^7^. However, there are some reports on reno-toxicity by statins^8^.

Furthermore, statins, HMG-CoA reductase inhibitors, a first-line therapy for dyslipidemia in diabetes, have been shown to improve diabetic nephropathy^9^,^10^,^11^.

Experimental studies have demonstrated beneficial effects of statins in diabetic nephropathy via reduced AGE accumulation and expression levels of RAGE, TGF-beta and MMP-9 in renal tissue^11^,^12^,^13^,^14^. Statins have also been suggested to delay the progression of the tubulointerstitial fibrosis in rats^15^. Moreover, reports indicate that long term use of statins does not result in any adverse effect on kidney tissue^11^,^16^. The renoprotective effects of statins could be due to their cholesterol-lowering properties^17^. However, statins were also shown to confer renal benefits in the Apo-E Knockout mouse with diabetes without affecting their circulating cholesterol and triglyceride levels, suggesting that it has cholesterol-lowering independent actions in reno-protection^18^. Additionally, statins have also been shown to exhibit both, cholesterol-lowering and cholesterol-lowering-independent effects on endothelial and vascular function^19^,^20^. Such findings led to several lines of investigations aiming towards the elucidation of cholesterol-independent “pleiotropic” effects of statins. In this regard, research on cancer revealed a novel cholesterol-lowering-independent action of statins as an inhibitor of histone deacetylase (HDAC) activity^19^,^21^. HDACs are a family of enzymes that balance the acetylation activities of histone acetyltransferases on chromatin remodeling and have essential roles in regulating gene transcription^22^. The novel potential of statins as HDAC inhibitors may be particularly relevant to diabetic nephropathy since diabetes has been associated with increase in HDAC activity in renal tissues, i.e., gene-specific transcriptional regulation was reportedly altered in diabetic kidneys via reducing acetylation of histone tails^23^,^24^,^25^. Moreover, HDAC inhibitors have been demonstrated to attenuate proteinuria, glomerulosclerosis, mesangial collagen deposition, oxidative-nitrosative stress and epithelial to mesenchymal transition in rodents with diabetes^24^,^25^,^26^,^27^,^28^. Nonetheless, in general, HDAC inhibitors did not influence blood glucose concentrations in rodents with diabetes. However, sodium butyrate (NaB), another HDAC inhibitor, significantly decreased plasma glucose levels besides showing beneficial effects on the diabetic kidney^29^.

Statins have been shown to be protective against renal disease in diabetic nephropathy, but the role of HDAC inhibition in this protection is far from clear. Moreover, a comparative evaluation of statins with non-statin cholesterol-lowering drugs on severity of diabetic nephropathy in experimental models has not been assessed. The present study addressed the above mentioned issues by using two mechanistically distinct means to lower circulating cholesterol, i.e., HMG-CoA reductase inhibition and GI-cholesterol binding on severity of diabetic nephropathy in streptozotocin induced diabetic nephropathy.

## Results

### Effect of Cholesterol-lowering drugs on diabetes

**Figure 1:**
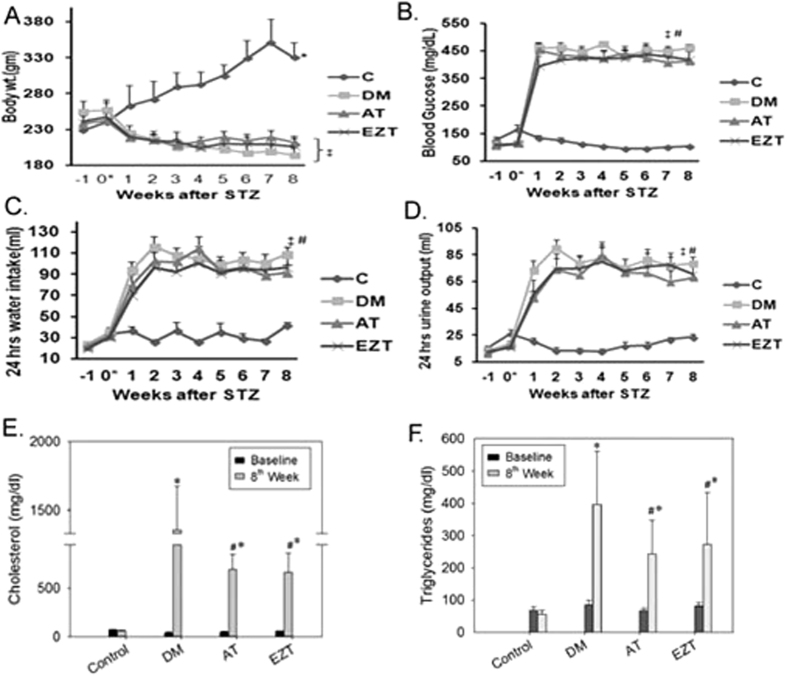
Diabetic features and lipid profile in control and diabetic rats (gavaged daily with either vehicle, AT or EZT), (n = 8–9/group). Figure shows change in (**A**) Body weight (**B**) blood glucose (**C**) 24 hrs water intake and (**D**) 24-hrs urine output during the course of the study. Panel (E) and (F) show total cholesterol and triglyceride levels, respectively in these rats at the end of 8^th^ week. Value are mean ± SEM, ^‡^p < 0.05 versus its own base line ^#^p < 0.05 versus control ^*^p < 0.05 versus DM by ANOVA. Abbreviations: C, control rats without diabetes; DM, vehicle treated rats with diabetes; AT, Atorvastatin treated DM rats; EZT, Ezetimibe treated DM rats.

We first examined diabetic parameters including dyslipidemia in the treated rats. STZ-induced diabetes significantly reduced serum insulin levels, and increased kidney weight, urinary glucose and protein levels compared to controls rats without diabetes (C) at the 8^th^ week (Table 1). Moreover, these parameters were similar in atorvastatin (AT)- and Ezetimibe- (EZT) treated STZ rats compared to untreated (DM) STZ rats. (Table 1, Fig. 1). Specific gravity of urine and red blood cell (RBC) levels in the urine were significantly higher in DM rats relative to control rats (C). The RBC levels were significantly lower in both treated groups, AT and EZT, relative to untreated DM rats. Specific gravity was however significantly lower only in AT treated group compared untreated DM.

Untreated DM rats had significantly elevated circulating cholesterol and triglycerides levels at the 8^th^ week of diabetes relative to control rats without diabetes. Both treatments (AT and EZT) significantly reduced cholesterol levels relative to untreated DM rats (Fig. 1). Moreover, the levels were reduced to a similar extent by both treatments. Furthermore, rats in both treatments (AT and EZT) had similar 24 hours food intake between themselves and compared to untreated diabetic rats (Supplementary Figure S1)

### Effect of Cholesterol- lowering drugs on HDAC activity in kidney tissue

**Figure 2:**
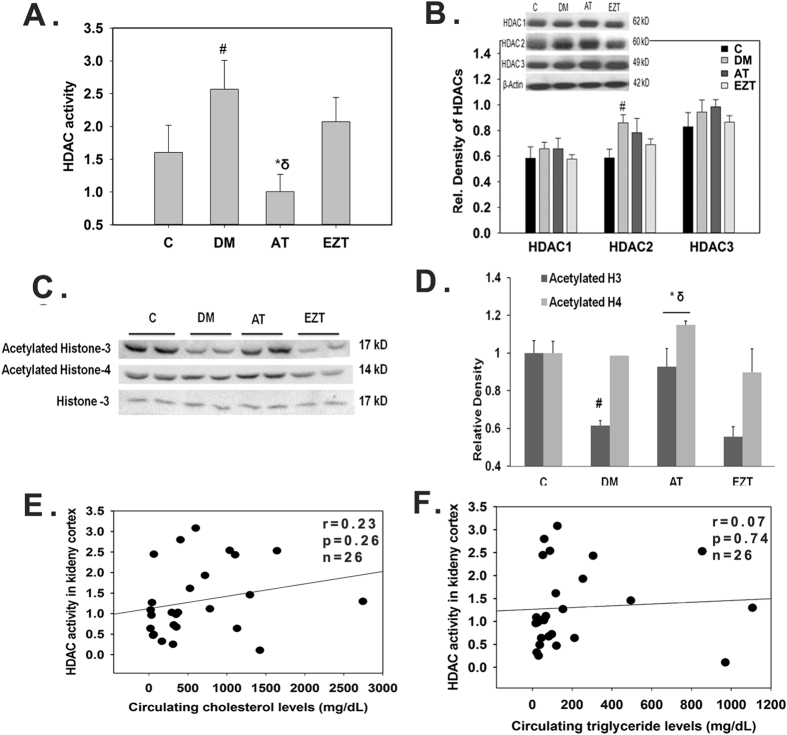
Figure shows (**A**) HDAC activity (n = 8–9 rats/group) and (**B**) HDAC protein levels in kidney cortex from control and diabetic rats (gavaged daily with either vehicle, AT or EZT) at the end of 8 weeks (3–5 rats/group). Representative lanes are shown from immunoblots of kidney cortex homogenates from 2–3 rats from each group run on the same gel. Each lane is loaded with the same amount of total protein from each rat. Multiple gels were run to accommodate to 3–5 rats from each group. The bar graph shows the densitometry summaries of the blots. For immunoblotting each membrane was cut at 55 KDa, the top portion (above 55 KDa) was probed with the antibody against HDAC1 and re-probed with HDAC2, and the bottom portion (below 55 KDa) was first probed with HDAC3 and re-probed with beta-actin. Panels (**C,D**), shows representative immunoblots for acetylated Histone levels along with densitometry summaries of the blots. For Immunoblotting each membrane was cut at 29 KDa first probed with antibodies against histone H3 (17 KDa) and further reprobe with antibodies against acetylated histone H3 (17 KDa) and H4 (14 KDa), and (**E,F**) shows Linear Regression plots between renal tissue HDAC activity and, circulating cholesterol and triglyceride levels, respectively in diabetic rats (untreated, AT treated or EZT treated) at the end of 8 weeks (n = 8-9/group). Value are mean ± SEM, ^#^p < 0.05 versus control *p < 0.05 versus DM, ^δ^p < 0.05 versus EZT by ANOVA. Abbreviations: C, control rats without diabetes; DM, vehicle treated rats with diabetes; AT, Atorvastatin treated DM rats; EZT, Ezetimibe treated DM rats.

We next examined the effects of AT and EZT on HDAC activity in the kidneys. Untreated DM rats had significantly higher HDAC activity in the renal cortex while AT treated rats had significantly lower renal HDAC among all the groups (Fig. 2A). HDAC activity in EZT treated rats, although lower, was not significantly different than untreated DM rats (Fig. 2A). Western blot analysis showed that HDAC protein expression of class I HDACs (1, 2 and 3) in kidney tissue remained similar in all groups at the end of study, except for HDAC2 levels which had a modest, but significant increase in DM rats compared to without diabetes controls (Fig. 2B). In addition, protein levels of class II HDACs i.e, HDAC 4 and 5 were also similar in kidney tissues from rats in each group at the end of study (Supplementary Figure S2). Moreover, AT treated rats had significantly more acetylated H3 and H4 levels relative to untreated DM rats (Fig. 2C,D).

Moreover, linear regression analysis revealed a weak and insignificant association between HDAC activity and circulating cholesterol and triglycerides (Fig. 2E and F). Thus cholesterol reduction alone might affect histone acetylation, at least in the kidney. In the next set of experiments, *in vitro* studies were performed to determined a direct effect of AT and/or EZT on histone acetylation and HDAC activity.

### Effects of Atorvastatin and Ezitimibe on H3 and H4 acetylation, and HDAC activity in primary mesangial cells

**Figure 3:**
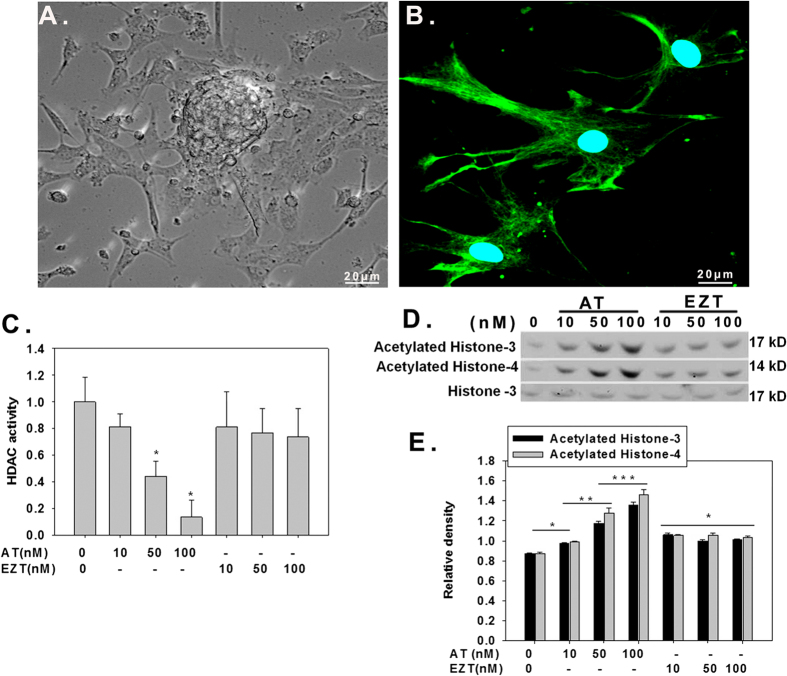
Effect of Atorvastatin (AT) and Ezetimibe (EZT) on HDAC activity and histone acetylation in primary glomerular mesangial cells. Panel (A) shows representative phase contrast image of cells at day 3^rd^ of culture. Panel (**B**) shows representative Confocal image of vimentin positive glomerular mesangial cells (green) and Hoechst nuclear stain (Blue) with at day 28^th^ of culture. Panel (C,D) shows HDAC activity and acetylated histones (H3 and H4) levels in these cells treated with vehicle or drugs ((10 nM to 100 nM) for 24 hours (n = 3 independent experiments). Total histone H3 was used as an internal control. (**E**) Shows the densitometry summaries of the immunoblots blot. For Immunoblotting each membrane was cut at 29 KDa first probed with antibodies against histone H3 (17 KDa) and further reprobe with antibodies against acetylated histone H3 and H4. Treatments was plotted as the mean ± SEM of three independent experiments; *p < 0.05 versus control **p < 0.05 versus 10 nM, ***p < 0.05 versus the effect of drug at 50 nM concentration by ANOVA.

To determine a direct effect of AT and/or EZT on Histone modulation we performed experiments using primary mesangial cells. We found that AT significantly increased H3 and H4 acetylation and inhibited HDAC activity in a dose- dependent manner in glomerular mesangial cells (Fig. 3). However, EZT did not seem to have any significant effect on histone acetylation or HDAC activity.

### Comparative effects of Atorvastatin and Ezetimibe on renal injury in rats with diabetes

**Figure 4:**
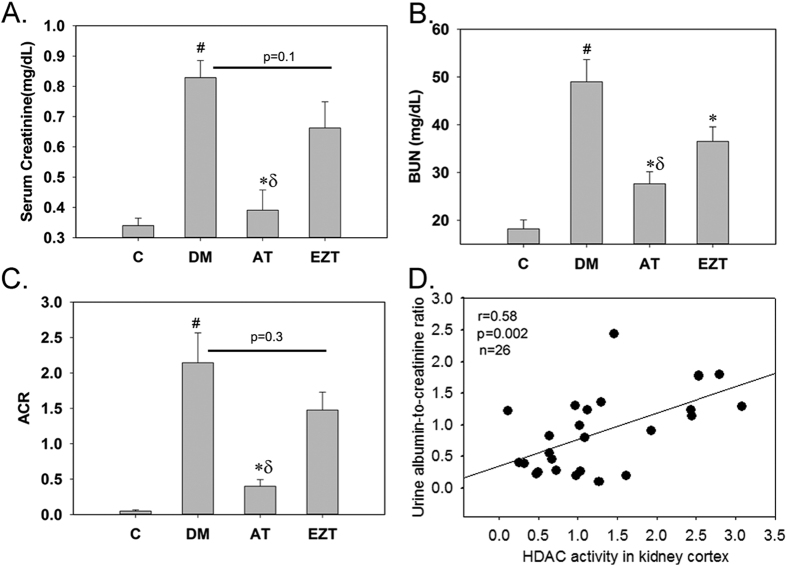
Figure shows (**A**) serum creatinine, (**B**) Blood Urea Nitrogen, BUN and (**C**) urine albumin-to-creatinine ratio, ACR in control and diabetic rats (gavaged daily with either vehicle, AT or EZT) at the end of 8 weeks. Panel (D) shows Linear Regression plots between renal tissue HDAC activity and urine ACR in diabetic rats (untreated, AT treated or EZT treated) at the end of 8 weeks (n = 8–9/group). Value are mean ± SEM, ^#^p < 0.05 versus control *p < 0.05 versus DM, ^δ^p < 0.05 versus EZT by ANOVA. Abbreviations: C control rats without diabetes; DM, vehicle treated rats with diabetes; AT, Atorvastatin treated DM rats; EZT, Ezetimibe treated DM rats.

We next compared the efficacy of AT and EZT with regard to attenuating the progression and severity of diabetic kidney injury. For this, renal fibrosis and urinary albumin-to-creatinine ratio (ACR), serum creatinine, and blood urea nitrogen (BUN) were evaluated. Rats with diabetes had significantly elevated urine ACR compared to without diabetes controls. Among the groups with diabetes, AT-treated rats had significantly lower urine ACR, serum creatinine and BUN (Fig. 4A–C). These parameters were also reduced in EZT rats, however, the reduction did not reached significance relative to untreated DM rats, except for BUN levels which were significantly reduced. To determine whether HDAC activity is related to renal injury in diabetic rats, a linear regression analysis was performed between ACR and renal tissue HDAC activity. The analysis revealed a strong positive relation between HDAC activity and urine ACR among all rats with diabetes in the study (Fig. 4D).

**Figure 5:**
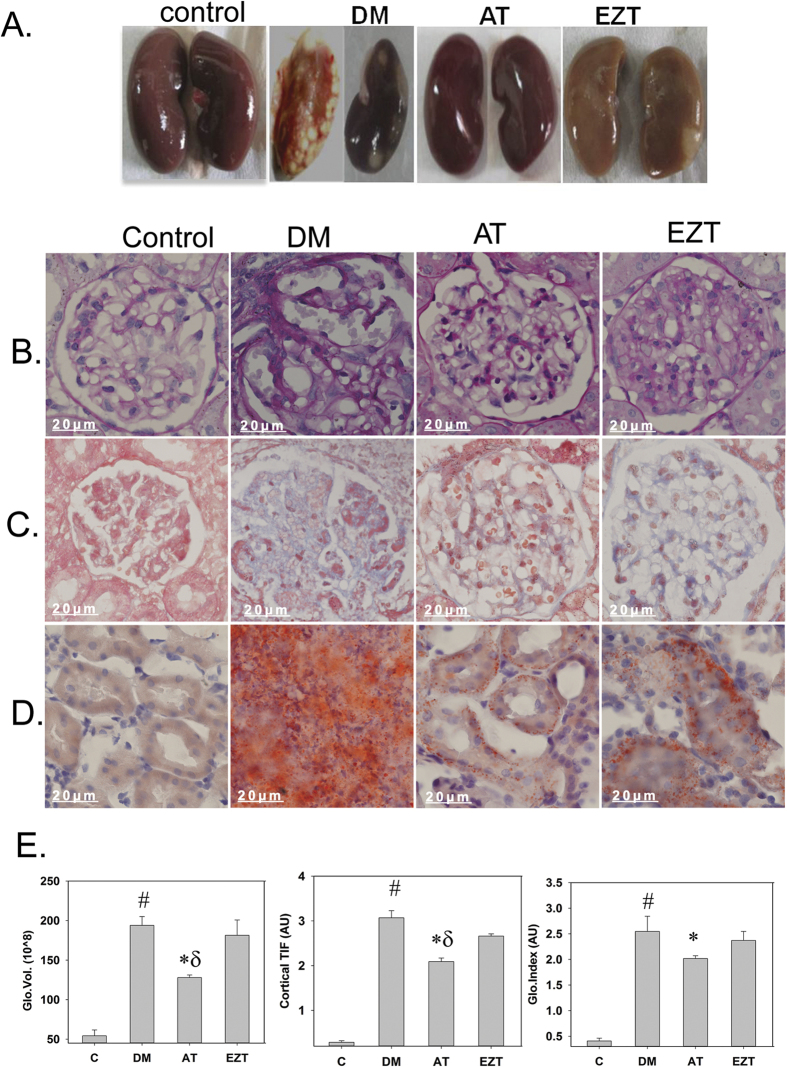
Effects of Atorvastatin and Ezetimibe on renal pathology in diabetic rats (gavaged daily with either vehicle, AT or EZT) at the end of 8 weeks (n = 8–9/group). Figure shows (**A**) kidney tissues from representative rat in each group. Panel (B–D) shows representative picture of kidney tissue section with stained Periodic acid-Schiff (**B**), Massion Trichome (**C**) and oil red (**D**) at (X600 magnification). Panel (E) shows histomorphometric analysis (n = 8/group); Glomerular Volume (Glo. Vol); cortical tubulointerstitial fibrosis index (Cortical TIF), and Glomerular (Glo. Index). Value are mean ± SEM, ^#^p < 0.05 versus control *p < 0.05 versus DM, p < 0.05 versus EZT by ANOVA. Abbreviations: C, control rats without diabetes; DM, vehicle treated rats with diabetes; AT, Atorvastatin treated DM rats; EZT, Ezetimibe treated DM rats.

Morphological analysis of kidney tissue revealed fibrosis in untreated DM rats (Fig. 5A). Among the treated groups, AT-treated rats showed relatively greater preservation of kidney morphology, as compared to the EZT-treated. Kidney tissue sections stained with PAS, MT, and oil red stains showed increased glomerular sclerosis (indicated by more glycogen deposition, pink staining in Fig. 5B), tubulointerstitial fibrosis (indicated by more collagen deposition, blue staining in Fig. 5C) and lipid deposition (indicated by more orange staining in Fig. 5D), respectively, in untreated DM rats compared to without diabetes controls (Fig. 5B–D). Moreover, both treatments attenuated the severity of the above pathology; however, only AT-treated rats had significantly reduced glomerular volume, cortical tubulointerstitial fibrosis, glomerulosclerosis index and lipid deposition compared to untreated DM rats (Fig. 5E). Results obtained using sirius red staining, for collagen deposition, revealed more collagen deposition in untreated DM rats (Supplementary Figure S3A). Both treatments had significantly lower collagen deposition compared to untreated DM rats. Moreover, AT-treated rats had significantly more reduction in collagen deposition compared to EZT. (Supplementary Figure S3B).

### Comparative effects of Atorvastatin and Ezetimibe on the expression of anti-fibrotic and fibrotic gene expression in rats with diabetes

**Figure 6:**
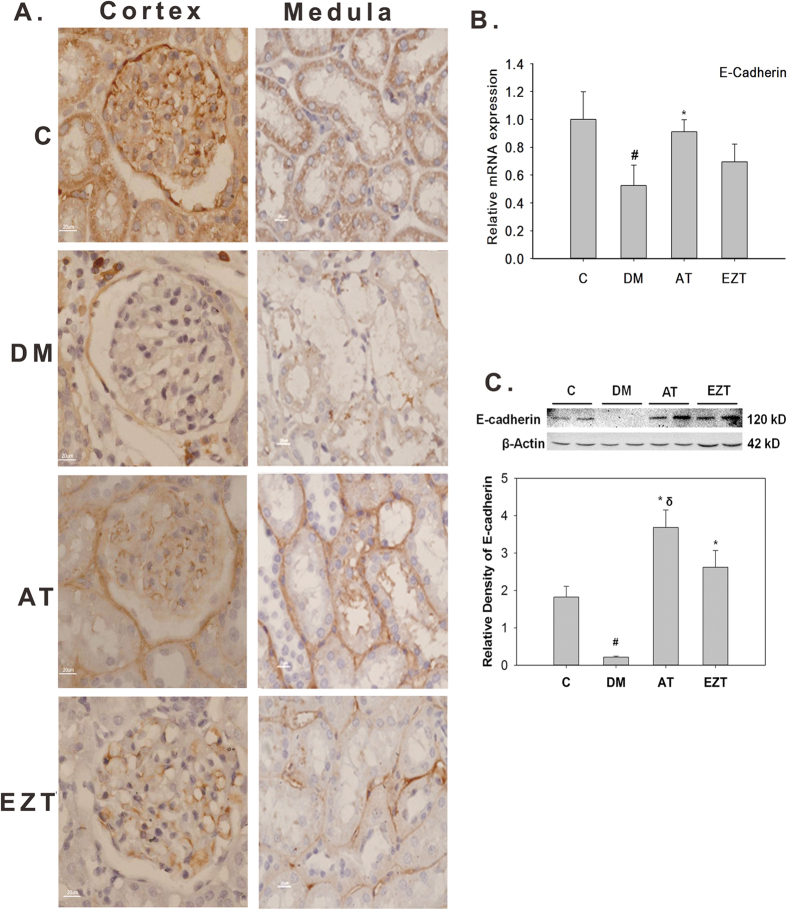
Effects of Atorvastatin and Ezetimibe on renal cortical expression of E-cadherin in control rats and diabetic rats (gavaged daily with either vehicle, AT or EZT) at the end of 8 weeks (n = 8–9/group). Figure (**A**) shows representative photomicrograph of E-cadherin protein at (X 600 magnifications), and (**B**) shows relative transcript level of E-cadherin using qRT-PCR analysis with GAPDH used as internal control. Value are mean ± SEM, ^#^p < 0.05 versus control *p < 0.05 versus DM by ANOVA. Abbreviations: C, non diabetic control rats; DM, vehicle treated diabetic rats; AT, Atorvastatin treated diabetic rats; EZT, Ezetimibe treated diabetic rats. (**C**) E-cadherin protein levels in kidney cortex from control and diabetic rats (gavaged daily with either vehicle, AT or EZT) at the end of 8 weeks (3–5 rats/group). Representative lanes are shown from immunoblots of kidney cortex homogenates from 2 rats from each group run on the same gel. Each lane is loaded with the same amount of total protein from each rat. Multiple gels were run to accommodate to 3–5 rats from each group. The bar graph shows the densitometry summaries of the blots. For immunoblotting each membrane was cut at 55 KDa, the top portion (above 71 kDa) was probed with the antibody against E-cadherin, and the bottom portion (below 71 kDa) was probed with beta-actin.

We next tested whether the two drugs differentially affected the expression of fibrotic and antifibrotic genes in kidney tissue. Immunohistochemistry revealed reduced expression of E-cadherin in the kidney tissue of untreated DM rats at the 8^th^ week of diabetes relative to without-diabetes controls (Fig. 6A). The expression levels were, however, similar between AT-treated rats and without-diabetes controls. This was further confirmed by qRT-qPCR analysis which showed a significantly lower transcript level of E-cadherin in DM rats relative to controls (Fig. 6B). Moreover, the mRNA expression was significantly higher only in AT-treated DM rats compared to untreated DM (Fig. 6B). Immunoblotting revealed similar regulation of E-cadherin at protein levels (Fig. 6C,D).

**Figure 7:**
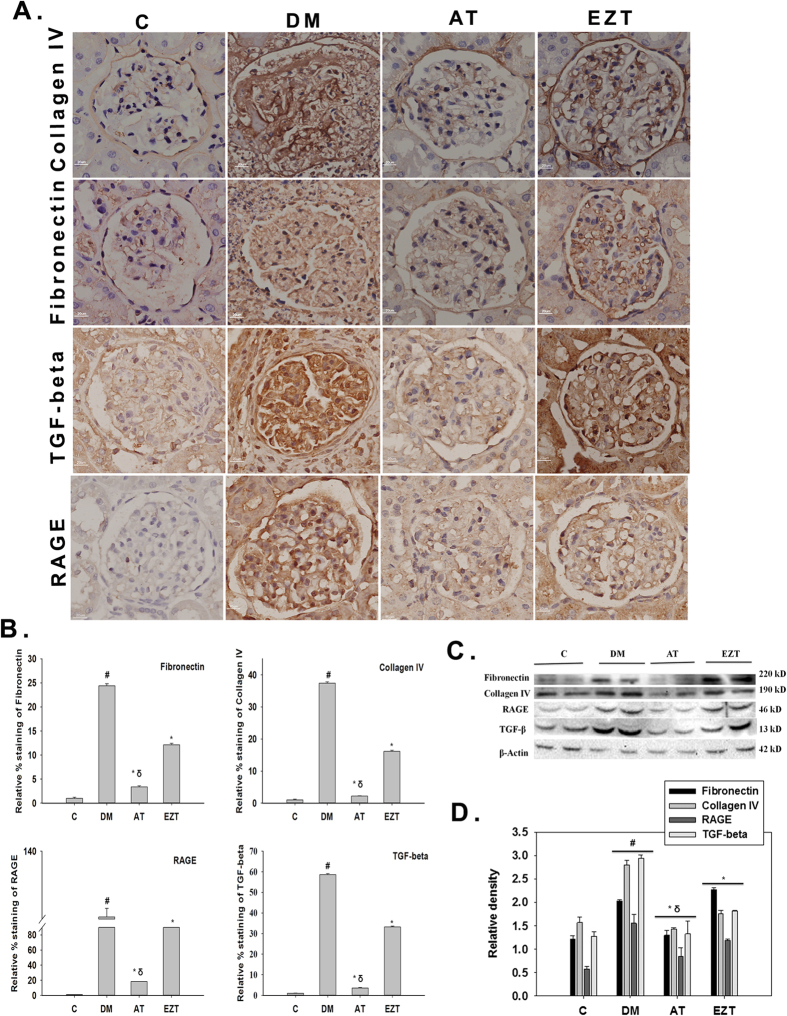
Effects of Atorvastatin and Ezetimibe on renal cortical protein expression of fibrotic genes (Collagen IV, fibronectin, TGF-beta, RAGE) in control and diabetic rats (gavaged daily with either vehicle, AT or EZT) at the end of 8 weeks (n = 8–9/group). Figure (**A**) shows representative photomicrograph at (X600 magnification); (**B**) shows semi-quantitative analysis of immunohistochemical staining. Pannel (C) and (D) show immunoblot and densitometry of fibrotic genes (Collagen IV, fibronectin, TGF-beta, RAGE). For Immunoblotting each membrane was cut at (71 KDa) and (29 KDa) first above 71 KDa probed with antibodies against fibronectin (220 KDa) and further reprobe with antibodies against collagen (190 KDa). Between cut blot was 71 KDa and 29 KDa probed with antibodies against RAGE (46 KDa) and beta actin (42 KDa). Representative lanes are shown from immunoblots of kidney cortex homogenates from 2 rats from each group run on the same gel. Each lane is loaded with the same amount of total protein from each rat. Multiple gels were run to accommodate to 3–5 rats from each group. Value are mean ± SEM, ^#^p < 0.05 versus control *p < 0.05 versus DM, ^δ^p < 0.05 versus EZT by ANOVA. Abbreviations: C, control rats without diabetes; DM, vehicle treated rats with diabetes; AT, Atorvastatin treated DM rats; EZT, Ezetimibe treated DM rats.

Immunohistochemistry revealed significantly higher protein expression of fibrotic genes such as, collagen (IV), fibronectin, TGF-β and RAGE in the kidney tissue of untreated DM rats relative to rats without diabetes (Fig. 7A and B). Furthermore, the expression of these proteins was significantly reduced in both AT- and EZT-treated groups compared to untreated DM rats (Fig. 7A and B). Between AT and EZT groups, AT had significantly lower expression of all these fibrotic markers, relative to the EZT group. Quantitative real time RT-PCR showed similar regulation of mRNA levels of these genes (Supplementary Figure 4A).

In addition, we also found significantly higher transcript levels of IL-6, IL-18, indicators of the inflammatory milieu^30^, and matrix metalloproteases (MMP-2 and 9) in the kidney tissue of untreated DM rats relative to rats without diabetes (Supplementary Figure 3B). The levels were significantly reduced in both, AT and EZT treated groups compared to untreated DM rats. AT-treated rats had significantly lower IL-18, MMP2 and MMP9 relative to EZT-treated rats (Supplementary Figure 4B).

### Transcriptional induction of anti-fibrotic gene in the kidney tissue of rats with diabetes via AT mediated HDAC inhibition

To establish whether the HDAC modulation observed was associated with transcriptional activity regulation of anti-fibrotic and fibrotic genes, we performed a native ChiP assay using antibodies against acetylated histones (H3 and H4).

**Figure 8:**
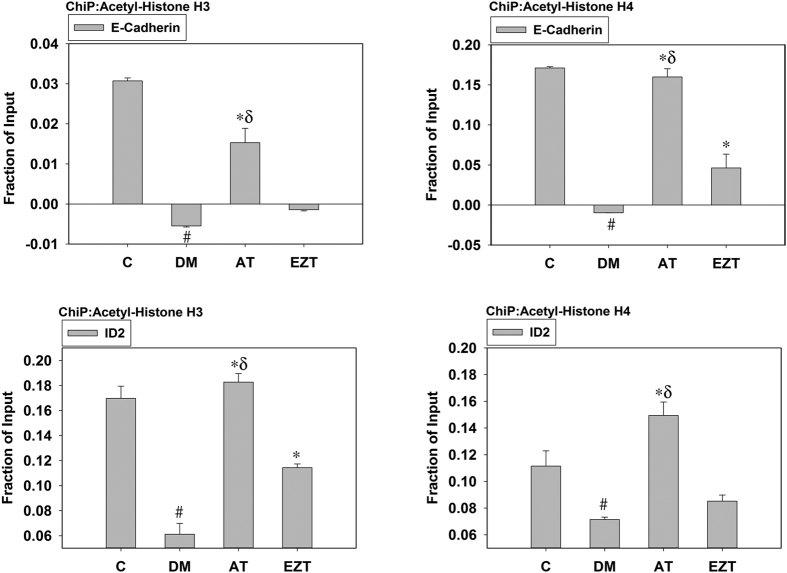
Effect of atorvastatin (AT) and ezetimibe (EZT) on transcriptional activity of anti-fibrotic genes in the kidney cortex from control and diabetic rats (gavaged daily with either vehicle, AT or EZT) at the end of 8 weeks (n = 8–9/group) using Chip assay. Figure shows chromatin Immunoprecipitation (ChiP) analysis of anti-fibrotic (E-cadherin, ID2) gene promoter regions using acetylated H3 and H4 antibodies. Value are mean ± SEM, ^#^p < 0.05 versus control *p < 0.05 versus DM, ^δ^p < 0.05 versus EZT by ANOVA. Abbreviations: C, control rats without diabetes; DM, vehicle treated rats with diabetes; AT, Atorvastatin treated DM rats; EZT, Ezetimibe treated DM rats.

We found acetylated H3 and H4 were significantly less associated with anti-fibrotic genes (E-cadherin and ID2, Fig. 8) in diabetic kidneys, suggesting transcriptional suppression of antifibrotic gene in DM rats. AT treatment markedly improved the association of both acetylated H3 and H4 with E-cadherin and ID2 chromatin, while these associations for the EZT-treated rats remained similarly repressed, as in the DM group.

In contrast acetylated H3 and H4 were significantly more associated with fibrotic genes in DM, relative to controls, suggesting transcriptional activation of fibrotic gene (Supplementary Figure S5). In the AT-treated group, however, these associations were significantly reduced relative to DM. Moreover, these associations were restored in AT-treated rats to levels similar to rats without diabetes. While, in the EZT treated rats, these associations were similar or significantly lower to DM rats.

## Discussion

The present study has addressed the following questions: 1) whether cholesterol-lowering by any means affects HDAC activity and chromatin modifications of fibrotic and anti-fibrotic genes in the diabetic kidney and, 2) if treatment with a specific statin confers any additional renal benefits beyond its cholesterol-lowering action in rats with diabetes (DM). These issues were addressed by comparing the effects of two mechanistically distinct cholesterol-lowering drugs, Atorvastatin, an HMG-CoA-reductase inhibitor and Ezetimibe, a non-statin, cholesterol-absorption inhibitor on the kidney of DM rats. We have demonstrated the following: (1) 8 weeks of therapy with either cholesterol-lowering drug, attenuated renal fibrosis, suggesting that cholesterol-lowering, alone likely can play an important role in improving renal pathology; (2) Atorvastatin imparts additional renal benefits over Ezetimibe despite reducing cholesterol levels to a similar extent, as indicated by significantly reduced urine ACR, serum creatinine, and BUN; (3) HDAC inhibition (along with increased H3 and H4 acetylation) and transcriptional induction of anti-fibrotic genes in AT rats’ kidney relative to vehicle treated DM. Gen-specific transcriptional regulation, i.e., post-translational modification of nucleosomal histone proteins by histone deacetylases (HDACs) may have a role in the genesis of nephropathy in subjects with diabetes^31^.

HDACs are a family of enzymes that regulate gene expression by attenuating the acetylation activities of histone acetyltransferases on chromatin remodeling^22^. Renal sclerosis in diabetes is associated with decreased expression of E-cadherin and increased expression of matrix proteins (collagen IV and fibronectin), TGF beta, and receptor for advance glycation end product (RAGE)^32^,^33^,^34^. Moreover, increased HDAC activity has been reported as a major regulator of diabetes associated renal injury^23^. In agreement with this idea, we found a significant increase in HDAC activity in untreated DM rats (Fig. 2A). In addition, the expression of the anti-fibrotic gene, E-cadherin, both at transcript and protein level, was significantly suppressed in the kidney cortex of these DM rats (Fig. 7). Moreover, AT treatment resulted in a significant reduction in HDAC activity and increased renal E-cadherin expression in DM rats (Figs 2A and 7). This led us to believe that the two events could be associated. Study by Peinado *et al*. 2004 support this hypothesis^35^. The authors have clearly demonstrated a role of HDAC activity in repression of the E-cadherin promoter by a transcription factor, “S*nail*”. HDAC inhibitors have been shown to play promising role in preventing fibrosis in liver, skin, lung^21^,^36^. In addition, HDAC inhibitors have also been shown to prevent epithelial to mesenchymal transition (EMT) in human REPTEC cells^24^. Thus, the additional renoprotective benefits by atorvastatin found in our study could be via its HDAC inhibitory action. However, only limited numbers of studies have investigated the histone modifying property of statins^21^,^37^.

Statins could modulate HDAC activity by altering the metabolic state of a cell. In this regard, conjugated CoA derivatives, including HMGCo-A has been sown to stimulate HDAC activity^38^. Our results are for the first time demonstrated HDAC inhibitory action of AT in kidney tissue. We also found that HDAC activity did not correlate with circulating cholesterol and triglyceride levels, and that Ezitimibe was not able to lower HDAC activity in DM rats (Fig. 2). Thus, AT by its HDAC inhibitory action may be imparting additional renal benefit over EZT. In support we showed a strong positive correlation of HDACs activity in renal cortex with urine ACR (Fig. 4C). Moreover, conventional HDAC inhibitors like trichostatin A and vorinostat have been shown to improve albuminuria and suppress epithelial to mesenchymal transition in models with diabetes^24^,^26^. Further, in the kidneys from AT treated rats acetylated H3 and H4 were associated with E-cadherin and ID2 promoters in significantly higher levels than in the kidneys from EZT treated rats or untreated DM rats (Fig. 8). The results again suggest transcriptional induction of anti-fibrotic genes via HDAC inhibition by AT. Our results could not identify the class of HDAC inhibited by AT as we have measured whole HDAC activity in our study. Nevertheless, findings from this study clearly demonstrated a direct role of AT in modulating H3 and H4 actelylation, and HDAC activity in renal mesangial cells (Fig. 3). Moreover, the increased expressions of fibrotic genes found in the kidney tissue of DM rats were prevented in AT, and also to some extent in Ezetimibe treated, DM rats (Supplementary Figure S5). Reduced HDAC activity is generally associated with transcriptional activation; therefore, it is not clear how these treatments led to transcriptional repression of fibrotic genes in our study. Although the mechanism remains unclear, transcriptional activation of repressors and their binding to the promoter of fibrotic genes and/or acetylation of non-histone proteins have been demonstrated to suppress fibrotic gene transcription^31^. Thus, we may speculate that anti-fibrotic genes may act as repressors for fibrotic gene transcription in AT treated rats’ kidneys in our study (Fig. 8 and Supplementary Figure S5). In this regard, study by Cho *et al*. 2010 has demonstrated the ability of E-cadherin gene to inhibit TGF-beta gene expression in rat liver cells^39^. At least what appears clear is that Atorvastatin treatment in our study normalized renal HDAC activity levels, or attenuated them in the direction of the without-diabetes state. Isoprenoid-dependent signaling could be one potential mechanisms for reduced fibrotic gene expression by atorvastatin in DM rats^40^. Thus, by inhibiting the biosynthesis of mevalonate statins could down-regulate fibrotic gene expression such as TFG-beta and fibrotectin via reduce the formation of GTP signaling molecules such as FPP^41^.

Additional studies would be needed to fully understand the precise histone modifications that occurred. Nevertheless, to the best of our knowledge, ours is the first report demonstrating a greater efficacy of Atorvastatin over Ezetimibe in attenuating renal pathology as indicated by significantly higher GV and GI score in the EZT relative to the AT group (Fig. 5), at least at the doses we tested. However, both AT and EZT impart kidney glomerular preservation in DM rats compared to untreated DM rats, despite no improvement in glucose homeostasis (Fig. 5). In agreement to our observation, AT and EZT has been reported to attenuate metabolic and renal dysfunction, independent of systemic glucose metabolism in both, animal model of diabetes and in subjects type 2 diabetes^42^,^43^,^44^. Thus, cholesterol-lowering per se, likely imparts reno-protection in conditions with diabetes. This renoprotection could be associated with the attenuation of high cholesterol-induced-oxidative stress^45^. In this regard, significant reductions in the renal pro-inflammatory cytokines IL-6 and IL-18 genes were found in AT and EZT groups, relative to untreated DM rats (Supplementary Figure 4). In addition, attenuation of oxidative stress has been suggested as a potential mechanism for renoprotection by other agents such as biotin and progesterone replacement in DM rats without affecting glucose metabolism^46^,^47^. However, it could be something entirely different that both drugs just happen to do, e.g., reduce BP, or just a coincidence that both drugs, by independent means, affect a particular parameter in a similar way.

Atorvastatin, in our study was superior to EZT with regard to attenuating urine ACR, BUN, and serum creatinine despite having similar cholesterol-lowering efficacy. Similar to our finding, studies by other investigators in rodents have also reported lowering of circulating cholesterol levels to a similar extent by AT and EZT using the same dose as used in our study^48^. However, a comparative study between Ezitimibe and Atorvastatin in dyslipidemic patients with CKD showed that atorvastatin was more potent in improving the serum lipid profile^49^. Several reasons may exist for this discrepancy. For example, human and rats may respond differently to the two drugs. Furthermore, the doses chosen in both ours and their study may have affected the final results^48^. In addition, both AT and EZT had no effect on blood glucose levels in our study. Moreover, AT and EZT at these doses were found to exert renoprotection in DM conditions^11^,^43^. Moreover, lipid-lowering-independent effects of Atorvastatin have been attributed for its better potency over conventional statins such as Rosuvastatin and Parvastatin, with regards to renoprotection in patients^50^,^51^. These studies strengthen our contention that Atorvastatin, in particular, may have protective actions above and beyond cholesterol-lowering.

Besides HDAC-inhibitory action of Atorvastatin, other pleiotropic effects such as its anti-inflammatory and anti-oxidant, could have also added for additional renal benefits by atorvastatin. In this regard, statins, by reducing the synthesis of mevalonate products, inhibit the activation of Rho and Ras guanosine triphosphatases that may influence various signaling pathways involving renal inflammatory, fibrogenic, proliferative, and cell-death responses. Statins also showed pleiotropic effect, by inhibiting the ERK1/2/NF-kappa B activation in endothelial and renal tubular epithelial cells^52^,^53^. Renal antioxidant effects with consequent endothelial function regulate the renal vasculature following statin treatment. This may also account for pleiotropic protection against renal injury^54^. Moreover, statins could inhibit macrophage infiltration in early phase of DN via its anti-inflammatory action^55^. In agreement we also found that diabetes associated increase in renal cortical TGF beta, RAGE, IL-6, IL-18, matrix metalloproteases (MMPs, MMP2 and 9) mRNA expression were prevented in treated groups. The prevention was, however, better in AT treated group. Our observations on MMPs are in agreement to studies showing attenuation of MMPs, important in remodeling and homeostasis of the extracellular matrix, by statins in experimental model with diabetes^56^,^57^.

Furthermore, statins has been shown to have blood pressure lowering affects both in humans and hypertensive model^58^,^59^,^60^,^61^. Therefore, Atorvastatin by blood pressure lowering, if any, may have accounted for the additional renoprotection. However, study based on a Japanese cohort of the Seven Countries Study has reported significant lowering in both, systolic as well as diastolic blood pressure by Ezitimibe treatment as well^62^. The blood pressure regulation by these treatments was, not however, evaluated in our study. Further studies are warranted to understand if the two treatments have differential effect on blood pressure lowering.

Overall we found greater efficacy of Atorvastatin versus Ezetimibe with regards to renoprotection in diabetes associated renal injury in rats. We suggest the possibility of transcriptional induction of anti-fibrotic and repression of fibrotic genes in diabetic kidney tissue via HDAC-inhibition as a major mechanism for this additional renoprotective effect.

## Material and Method

### Animal

All experiments were performed in accordance Institutional Animal Ethics Committee, IAEC (Ref. No. PGI/EP/AEC/17/2015) which is consistent with Committee for the Purpose of Control and Supervision of Experiments on Animals (CPCSEA, New Delhi, India) guidelines. Experimental protocols were approved by Sanjay Gandhi Postgraduate Institute of Medical Sciences, Lucknow (CEPSCEA Registration # 57/PO/ReBi/SL/99/CEPSEA). Male Wistar rats at 200–300 g were fed with standard animal chow diet and had free access to tap water ad libitum. Rats (N = 26) were made diabetic via intraperitoneal administration of streptozotocin (50 mg/kg body weight) dissolved in 0.1 M citric acid buffer with PH 4.5 after 18 hrs fasting^63^ (Sigma Chemical, St. Louis, MO). Tail blood samples were used to monitor blood glucose levels after 48 hours of injection using a glucometer (Abbott Diabetes Care Inc. Alameda, CA,) and rats were classed as diabetics when glycemia exceeded 11 mmol/L (1 mmol = 18 mg glucose)^64^. After 3 days of STZ injections, rats were randomized (n = 8–9/group) to receive one of the three treatment via gavage: 1) Vehicle, DM; 2) atorvastatin (Cipla Ltd. Mumbai, India), an HMG-CoA reductase inhibitor (20 mg/kg body weight0, AT; 3) Ezetimibe (Sigma Chemical, St. Louis, MO), a cholesterol-binding drug (5.0 mg kg^**−1**^ body weight), EZT. All rats received a diet supplemented with 4% cholesterol and 1% cholic acid (Sigma Chemical, St. Louis, MO) to increase the severity of diabetic nephropathy. Control rats (n  = 8) were injected with an equal volume of citrate buffer and were fed regular chow diet. Food and water intake was monitored, and a 24-hours urine collection was done using metabolic cages (Lab Product Inc. Seaford, DE). After eight weeks, rats were euthanized under Isoflurane. The left kidneys were perfusion fixed for histological analysis.

### Biochemical analysis

Serum was analyzed for total cholesterol and triglycerides (Siemens Autopack kit) and insulin using an EIA kit (Spi bio^(R)^ bertin pharma Insulin,). For urine analysis, 24-hour urine samples were centrifuged at 4,000 rpm for 10 min to remove cell debris. Urine albumin was measured using an ELISA (Bethyl Laboratories, Montgomery, TX) and creatinine by a modified Jaffe’s method (Span Diagnostics, Gujarat, India). Other qualitative analyses were done using Uro Colour ^TM^ 10 reagent strips (Standard Diagnostic, INC).

### HDAC activity Assay

Histone Deacetylase (HDAC) activity was assayed using the Fluor-de-Lys™ HDAC Fluorimetric Activity Assay Kit (Enzo Life Sciences) as reported previously^21^. Briefly, tissue homogenates (25 μg of total protein) from kidney cortex and cell lysates and cell lysates were incubated with Fluor-de-Lys substrate in triplicates for 30 min at 37 °C to initiate the HDAC reaction^65^. Fluor-de-Lys Developer was then added, and the mixture was incubated for another 10 min at room temperature. Fluorescence intensity was measured in a fluorometer Synergy XT with an excitation wavelength of 360 ± 40 nm and an emission wavelength of 460 ± 40 nm.

### Chromatin immunoprecipitation (ChiP) Assay

Histone ChIPs were performed using a native ChIP protocol. The procedure consists of five steps: nuclei isolation from kidney cortex; fragmentation of chromatin using Mnase; purification of nucleosomes using HAP; immunoprecipitation of modified Nucleosomes using antibodies against anti-acetyl Histone H3 (Abcam MA USA) and anti-acetyl Histone H4 (Millipore); and qPCR analysis of DNA associated with modified histones^66^. We used 15 μg of chromatin for the chip assay. qPCR was performed on ABI 7900 thermal cycler (Applied Biosystems,) using promoter-specific primers. The promoter specific primers for rat were: E-cadherin, TCTAGGAACTTATTACGCCATTCC(F) and AGCTGGGAGACCAGAGATAATA(R); ID2, GTCACTGAGTTTCCGAGAAGG(F) and GGGAAACCTACCCACATACAC(R); collagen-CTTGCGGAGTGACCAAAGT(F) and ATCGGAGCTGGAGGAATCA(R); fibronectin-TGTCTGTGTCTGTGTCTGTG(F) and CTGGGAGATGTCCATCTGTG(R); TGF-β- GTTTGAAGGATCCAGAGAGAGG(F) and GGAAGGAGAGAAAGGAAGTCAG(R); RAGE(F)TCACTCAGAAAGACAACAGACC (F) RAGE(R)CAGCTCACTAGTGCTCTATCTTG (R). ChiP qPCR signals are presented as a percentage of input.

### Morphology

Three μm kidney tissue sections were stained with Periodic Acid and Schiff, PAS, for glycogen deposition; Massion Trichome, MT, and Sirious red for collagen deposition, and oil red for renal lipid accumulation (Sigma, St Louis, MO)^67^. PAS-stained sections were examined for the degree of glomerular damage (*Glomerulosclerotic Index)* and MT stained sections for the degree of tubulointerstitial fibrosis using using a Nikon Eclipse E600 light microscope by a semi-quantitative scoring method as described by Maric *et al*. 2004^68^. Sirious red and Oil red O (Sigma-Aldrich, St. Louis, MO) quantified by image J software^69^,^70^.

### Glomerulosclerotic Index (GSI index)

Randomly selected glomeruli per section (~100) were assessed using a semi-quantitative scoring method; grade 0, normal glomeruli; grade 1, sclerotic area up to 25% (minimal sclerosis); grade 2, sclerotic area 25 to 50% (moderate sclerosis); grade 3, sclerotic area 50 to 75% (moderate-severe sclerosis); grade 4, sclerotic area 75 to 100% (severe sclerosis). The GSI was calculated using equation 1 (Eq. 1) given below


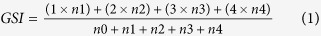


where n is the number of glomeruli in each grade of glomerulosclerosis^68^.

### Glomerular Volume (GV)

GV was estimated using Image as previously described method^71^. The mean glomerular volume was determined from the mean glomerular cross sectional area on the basis of the average area of 20 glomeruli in each group. For GV the following equation (Eq. 2) was used:


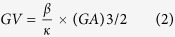


(β  = 1.38, the shape coefficient of spheres (the idealized shape of glomeruli), and k = 1.1, the size distribution coefficient and GA; glomerular area)^72^.

### Tubulointerstitial Fibrosis

The degree of tubulointerstitial fibrosis was graded on a scale of 0 to 4: grade 0, affected area 0% (normal); grade 1, affected area (tubular atrophy or dilatation, deposition of ECM, and interstitial cell proliferation) less than 10%; grade 2, affected area 10 to 25%; grade 3, affected area 25 to 75%; grade 4, affected area greater than 75%^68^.

### Immunohistochemistry

Three μm thin sections of the kidney tissue blocks were used for immunohistochemical analysis using a standard method as described previously^73^,^74^,^75^. Briefly the tissue sections were incubated with 10% non-immune goat serum followed by primary antibodies against fibronectin IST-9, collagen IV or TGF beta 1 from Abcam Inc or with Rage or E-cadherin from Santa Cruz Biotechnology (Santa Cruz, CA) Images were captured using a Nikon Eclipse E600 light microscope and quantified by image J software with IHC profiler plugin as described by Varghese *et al*. 2014^76^.

### RNA Isolation and Real-Time qPCR

Total RNA (500 ng) from rat Kidney cortex was subjected to RT-qPCR using SYBR-green PCR Master Mix (Applied Biosystems) as described previously^74^,^75^,^76^,^77^,^78^ qPCRs using SYBR green reagent with gene-specific primers and GAPDH gene primers (internal control). The gene specific primers for rat were: E-cadherin- GCCCAGGAAATACACCCCTC (F) and ACTCAGGTCCAAATCAGCCG (R); collagen IVα- GGAGAACAAGGGGTCAGTGG (F) and TCCTGTTGGGGCAAAGTCTC (R); fibronectin- CCTGTGTTCTCCCGTTTCACT (F) and TGTGCTACACCACAGATGTCC (R); TGF-β- TCCCAACTACAGAAAAGCAGTCA (F) and GCAATGCAGACGAAGCAGAC (R); IL6- CCCAACTTCCAATGCTCTCCT (F) and GGATGGTCTTGGTCCTTAGCC (R); IL-18- GAACTGAGCCCCTCCCTACTA (F) and TGTCTTCAATCCATCCCAGAGC (R); MMP-2- AAGGATGGAGGCACGATTGG (F) and GGGAACTTGATGATGGGCGA (R); MMP-9- ATGGTTTCTGCCCCAGTGAG (F) and CCTTTAGTGGTGCAGGCAGA (R); RAGE-GAGGATGAGGGCATCTACAGC (F) and TCACCGGTTTCTGTGACCC (R), and GAPDH gene primers (AGGTCGGTGTGAACGGATTTG(F) and TGTAGACCATGTAGTTGAGGTCA (R). Fold changes in gene expression were calculated using the 2^−ΔΔCT^ method.

### Glomerular Mesangial Cell Culture

Isolation and characterization of primary glomerular mesangial cells was performed as described by Wilson and Stewart 1983^79^. Briefly, kidneys cortex from male wistar rat at 3 month of age, isolated in aseptic conditions, were placed in a sterile Petri dish with RPMI-1640 wash medium containing L-glutamine, penicillin, streptomycin and amphotericin B. 1–2 mm^2^ chopped tissues pieces were digested with collagenase and a passed through a series of stainless steel sieves to retains the glomeruli. Rinsed glomeruli were collected and transferred at a concentration of approx 15–20 glomeruli/ml to a fibronectin-coated culture flask. The flasks were incubated at 37 °C in a 5% CO2 incubator by replacing media at every 4–5 days. The cells were fully characterized between 4–8 passages. To determine the effect of drugs cells were seeded in to 6-well cell culture plate and incubated overnight at 37 °C. Thereafter, the culture supernatant was replaced with different concentration (10 nM, 50 nM and 100 nM) of atorvastatin or Ezitimibe or vehicle (only medium) containing culture medium in triplicates and incubated at 37 °C for 24 h. After incubation cells were harvested for for HDAC activity, and for immuno-blotting of acetylated histones.

### Immunofluorescence

Immunostaining of mesangial cells were carried out at day 28^th^ of culture. as described by us previously^74^. Briefly, fixed and permeabilized cells were pre-incubated with PBS containing 1% BSA for 1 h at 37 °C followed by overningt incubation (at 4 °C) with mouse anti-vimentin antibodies (Invitrogen). In addition, mouse anticytokeratin (pan), clone AE1/AE3 antibodies (Invitrogen) was used as epithelial cells marker or only PBS with 1% BSA was used in place of primary antibodies as negative control. After PBS washing, cells were incubated for 1 h with rabbit anti-mouse IgG FITC conjugate (diluted 1:50 in PBS containing 1% BSA), washed and mounted in VECTASHIED mounting medium (Vector Laboratories, Burlingame, CA) and observed under Confocal microscope (OLYMPUS, FV10i).

### Western Blotting

Protein levels from rat kidney cortex homogenates and from cell lysates were quantitated using immunoblotting as described by us previously^73^,^74^,^75^,^76^,^77^,^78^,^79^,^80^. The antibodies against HDACs (Cell signalling technology, MA USA), E-cadherin, Fibronectin, Collagen, RAGE (Santa Cruz Biotechnology, USA), TGF-beta, Histone H3, acetyl Histone H3 (Abcam MA USA) and anti-acetyl Histone H4 (Millipore) and, anti-β-actin (Cell signalling technology, MA USA) were used and signals were detected using a chemi-luminiscence-based detection system (Amersham). The levels of protein expression were quatitated using the Quantity one (BioRad) software.

### Statistical Analyses

Data are expressed as means ± SEM. Comparisons were made using ANOVA. Linear regression analysis was used to assess the relationship of renal HDAC activity with lipid levels, and with urine albumin-to-creatinine ratio among all DM rats. Statistical analysis was done using Sigma Plot 12.3 (Chicago, IL). P values < 0.05 were considered significant.

## Additional Information

**How to cite this article**: Singh, R. S. *et al*. Greater efficacy of atorvastatin versus a non-statin lipid-lowering agent against renal injury: potential role as a histone deacetylase inhibitor. *Sci. Rep.* **6**, 38034; doi: 10.1038/srep38034 (2016).

**Publisher's note:** Springer Nature remains neutral with regard to jurisdictional claims in published maps and institutional affiliations.

## Supplementary Material

Supplementary Figures

## Figures and Tables

**Table 1:** Diabetes was induced in male Wistar rats by intraperitoneal injection of streptozotocin (STZ) at 50 mg/kg body weight in sodium citrate buffer.

Variable	C (N = 8)	DM (N = 8)	AT (N = 9)	(EZTN = 9)
(TK wt./Body wt)*100	0.835 ± 0.037	1.27 ± 0.084#	1.29 ± 0.058#	1.21 ± 0.06#
Insulin (ng/ml)				
0 weeks	4.19 ± 0.32	13 ± 0.45	3.49±0.44	25 ± 0.40
8 weeks	4.7 ± 0.52	0.085 ± 0.02‡#	0.062 ± 0.016‡#	0.088 ± 0.027‡#
Urine Analysis				
Urinary Glucose (mg/dL)	0.0±0.0	2000 ± 0.0#	2000 ± 0.0#	2000 ± 0.0#
Urinary Protein (mg/dL)	0.0 ± 0.0	21.66 ± 5.13#	5.63 ± 1.28#*	12.3 ± 2.57#*
Urinary (RBC/μl)	0.0 ± 0.0	153.33 ± 26#	86.87 ± 28.6#*	102.3 ± 28.7#*
Urinary PH	7.11±0.11	6.66 ± 0.14	6.68 ± 0.12	6.69 ± 0.10
Specific Gravity	1.019 ± 0.002	1.029 ± 0.002#	1.023 ± 0.002*	1.027 ± 0.002#

Rats were gavaged daily with either vehicle (DM), Atorvastatin (AT) (20 mg/kg body wt) or Ezetimibe (EZT) (5 mg/kg Body weight). All rats had unrestricted access to food/water and were maintained for 8 weeks. Table shows physical and biochemical parameters at the end of 8 weeks. Value are mean ± SEM. ^‡^p < 0.05 versus its own base line. ^#^p < 0.05 versus control and *p < 0.05 versus DM by ANOVA. Abbreviations: C, control rats without diabetes; DM, vehicle treated rats with diabetes; AT, Atorvastatin treated DM rats; EZT, Ezetimibe treated DM rats; TK wt./Body wt, ratio of total kidney weight to whole body weight.
