# Supplementary material for: Greater efficacy of atorvastatin versus a non-statin lipid-lowering agent against renal injury: potential role as a histone deacetylase inhibitor
Source: Sci Rep. 2016 Nov 30;6:38034. doi: 10.1038/srep38034 (PMC5128790; doi:10.1038/srep38034)
Supplement: Supplementary Figures [file srep38034-s1.pdf]

Greater efficacy of atorvastatin versus a non-statin lipid-lowering agent against renal injury: potential role as a histone deacetylase inhibitor

Ravi Shankar Singh[1], Dharmendra Kumar Chaudhary[1], Aradhana Mohan[1], Praveen Kumar[1,] Chandra Prakash Chaturvedi[2], Carolyn M. Ecelbarger[3], Madan M. Godbole[1] and Swasti Tiwari[1*]

[1]Department of Molecular Medicine & Biotechnology, Sanjay Gandhi Post Graduate Institute of Medical Sciences, Lucknow, India.

[2]Department of Hematology, Sanjay Gandhi Post Graduate Institute of Medical Sciences, Lucknow, India.

[3]Department of Medicine, Georgetown University, Washington D.C., USA.

Corresponding author

Dr. Swasti Tiwari,
Associate Professor & Head
Department of Molecular Medicine and Biotechnology
Sanjay Gandhi PGI,
Lucknow-226014
Phone: +91-8004904827
E-mail: tiwaris@sgpgi.ac.in

Supplementary Figure S1

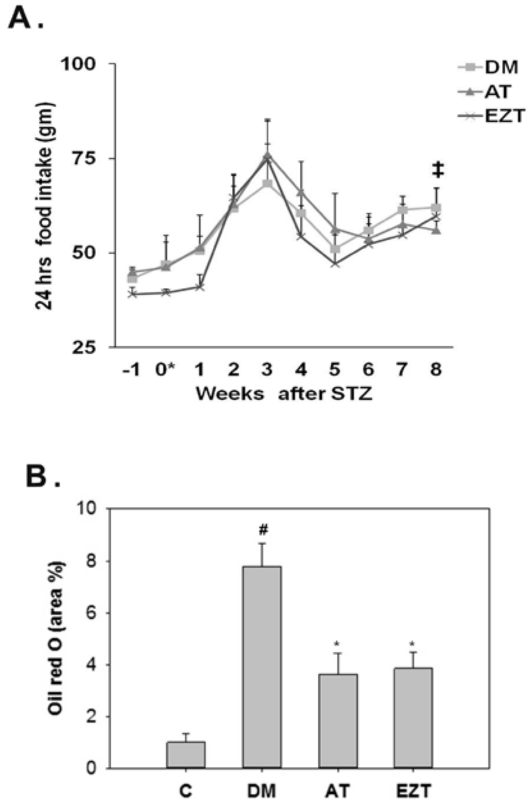

Supplementary Figure S1: Effects of Atorvastatin and Ezetimibe on Food intake and lipid deposition in diabetic rats (gavaged daily with either vehicle, AT or EZT) (n=8-9/group). Figure (A) shows food intake and figure (B) shows quantitation of oil red. Value are mean ± SEM, ‡p< 0.05 versus its own base line #p< 0.05 versus control *p< 0.05 versus DM by ANOVA. Abbreviations: C, control rats without diabetes; DM, vehicle treated rats with diabetes; AT, Atorvastatin treated DM rats; EZT, Ezetimibe treated DM rats.

Supplementary Figure S2

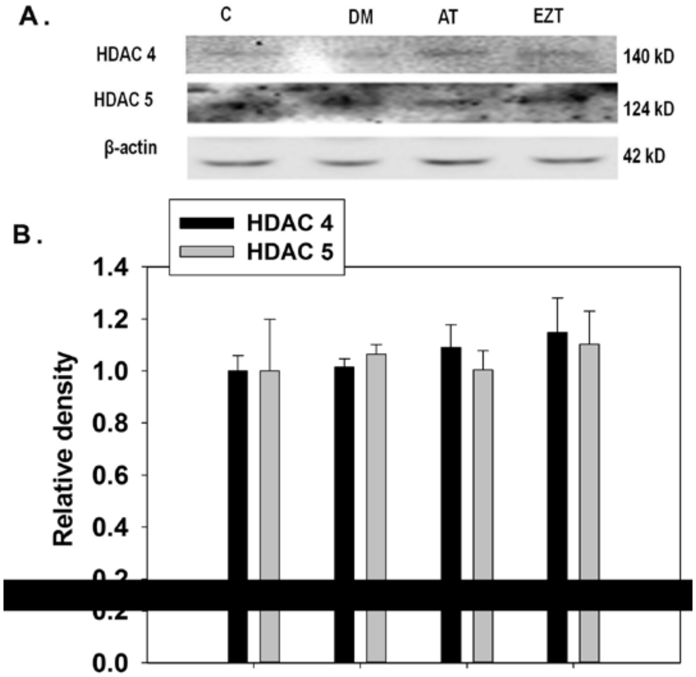

Supplementary Figure S2: Figure shows class II HDACs (4 and 5) protein levels in kidney cortex from control and diabetic rats (gavaged daily with either vehicle, AT or EZT) at the end of 8 weeks (n=3-5 rats/group). Representative lanes are shown from immunoblots of kidney cortex homogenates of one rats from each group run on the same gel. Each lane is loaded with the same amount of total protein from each rat. Multiple gels were run to accommodate to 3-5 rats from each group. The bar graph shows the densitometry summaries of the blots. For immunoblotting each membrane was cut at 71 KDa, the top portion (above 71 KDa) was probed with the antibody against HDAC4 and re-probed with HDAC5, and the bottom portion (below 71 KDa) was with beta-actin.. Value are mean ± SEM, $^{\#}p< 0.05$ versus control *p< 0.05 versus DM, $^{\delta}p < 0.05$ versus EZT by ANOVA.Abbreviations: C, control rats without diabetes; DM, vehicle treated rats with diabetes; AT, Atorvastatin treated DM rats; EZT, Ezetimibe treated DM rats.

Supplementary Figure S3

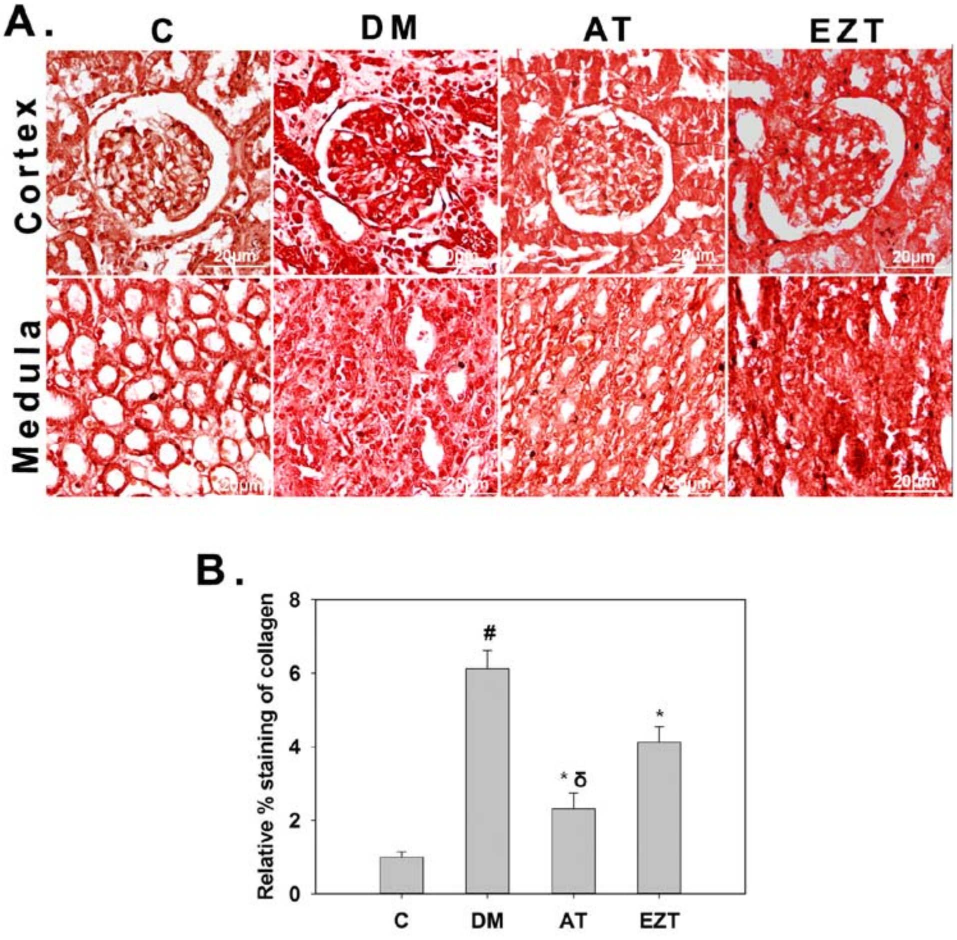

Supplementary Figure S3 Effects of Atorvastatin and Ezetimibe on collagen deposition in kidneys from diabetic rats (gavaged daily with either vehicle , AT or EZT) at the end of 8 weeks (n=3/group). Panel (A) shows representative picture of kidney tissue section with stained Sirius red stain at (X400 magnification). Panel (B) shows quantitation of Sirius red staining using NIH image J software (n=3/group). Abbreviations: C, control rats without diabetes; DM, vehicle treated rats with diabetes; AT, Atorvastatin treated DM rats; EZT, Ezetimibe treated DM rats.

Supplementary Figure S4

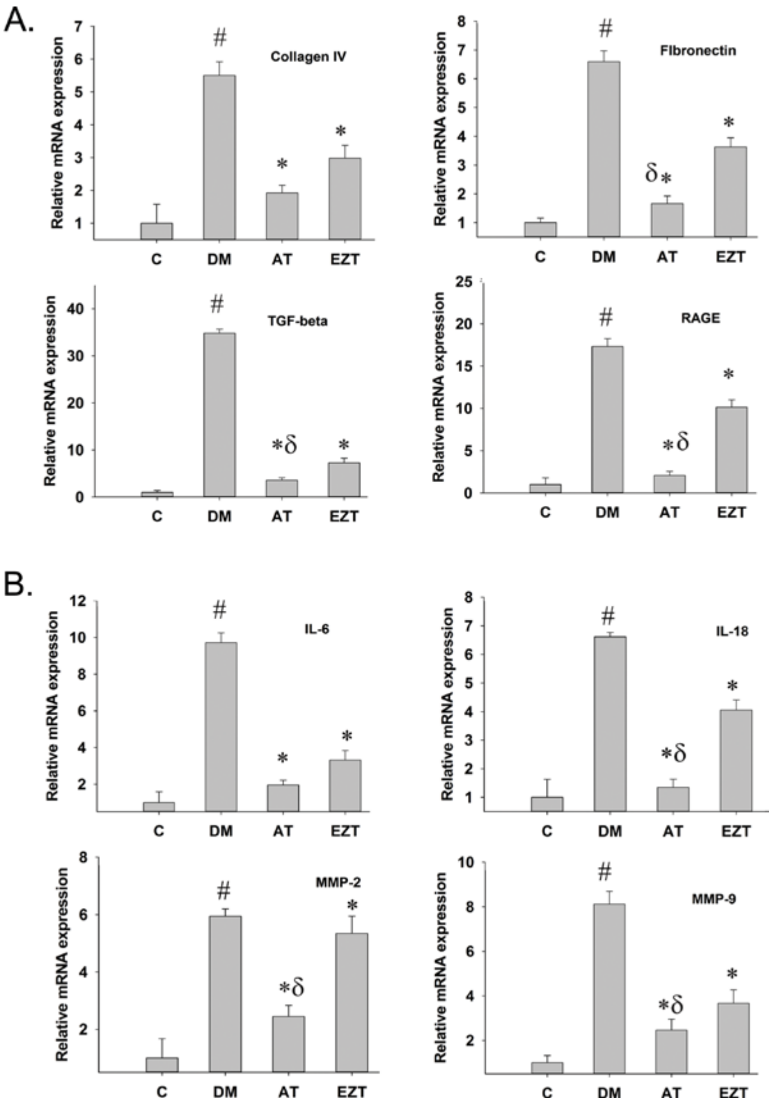

Supplementary Figure S4: Effects of Atorvastatin and Ezetimibe on transcript levels of (A) fibrotic genes, and (B) inflammatory cytokines (IL-6 and IL-8) and matrix metalloproteinase (MMP-2 and 9) in the kidney cortex from control and diabetic rats (gavaged daily with either vehicle , AT or EZT) at the end of 8 weeks (n=8-9/group) using qRT-PCR analysis with GAPDH as internal control. Value are mean ± SEM, $^{\#}p< 0.05$ versus control $*p< 0.05$ versus DM, $^{\delta}p < 0.05$ versus EZT by ANOVA. . Abbreviations: C, control rats without diabetes; DM, vehicle treated rats with diabetes; AT, Atorvastatin treated DM rats; EZT, Ezetimibe treated DM rats.

Supplementary Figure S5

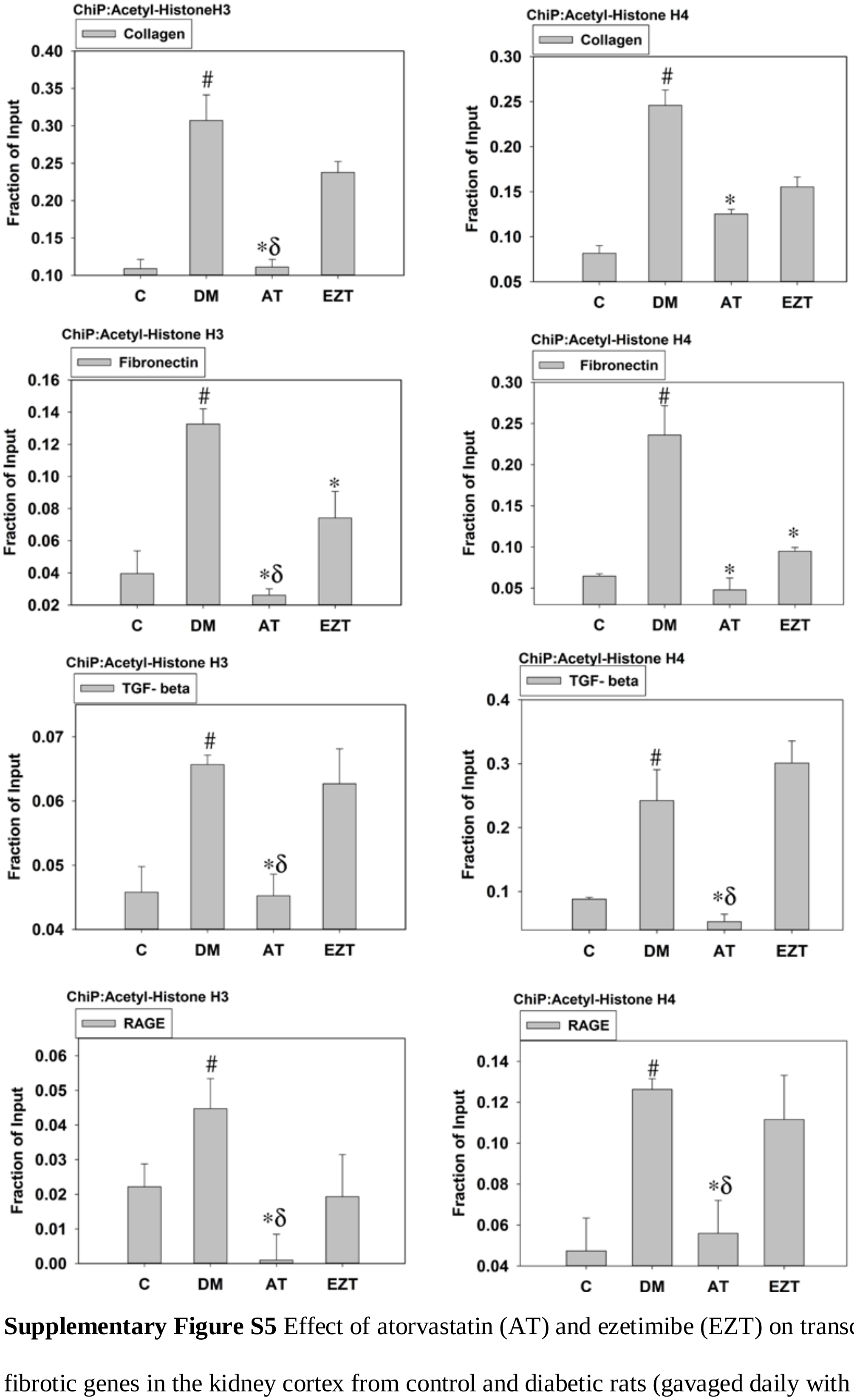

Supplementary Figure S5 Effect of atorvastatin (AT) and ezetimibe (EZT) on transcriptional activity of fibrotic genes in the kidney cortex from control and diabetic rats (gavaged daily with either vehicle , AT

or EZT) at the end of 8 weeks (n=8-9/group) using Chip assay. Figure shows chromatin Immunoprecipitation (ChiP) analysis of fibrotic gene (collagen, fibronectin, TGF-beta and RAGE) promoter regions using acetylated H3 and H4 antibodies. Value are mean ± SEM, $^{\#}p < 0.05$ versus control $^{*}p < 0.05$ versus DM, $^{\delta}p < 0.05$ versus EZT by ANOVA. . Abbreviations: C, control rats without diabetes; DM, vehicle treated rats with diabetes; AT, Atorvastatin treated DM rats; EZT, Ezetimibe treated DM rats.
